# Ribosome-Associated ncRNAs (rancRNAs) Adjust Translation and Shape Proteomes

**DOI:** 10.3390/ncrna8020022

**Published:** 2022-03-10

**Authors:** Valentina Pecoraro, Alessia Rosina, Norbert Polacek

**Affiliations:** 1 Department of Chemistry, Biochemistry and Pharmaceutical Sciences, University of Bern, 3012 Bern, Switzerland; valentina.pecoraro@unibe.ch (V.P.); alessia.rosina@unibe.ch (A.R.); 2 Graduate School for Cellular and Biomedical Sciences, University of Bern, 3012 Bern, Switzerland

**Keywords:** translation control, stress response, ribosome functions, non-protein coding RNA

## Abstract

The regulation of protein synthesis is of extreme importance for cell survival in challenging environmental conditions. Modulating gene expression at the level of translation allows a swift and low-energy-cost response to external stimuli. In the last decade, an emerging class of regulatory ncRNAs, namely ribosome-associated non-coding RNAs (rancRNAs), has been discovered. These rancRNAs have proven to be efficient players in the regulation of translation as a first wave of stress adaptation by directly targeting the ribosome, the central enzyme of protein production. This underlying principle appears to be highly conserved, since rancRNAs are present in all three domains of life. Here, we review the major findings and mechanistic peculiarities of rancRNAs, a class of transcripts that is providing new and broader perspectives on the complexity of the ribosome and translation regulation.

## 1. Introduction

With the implementation of avant-garde technologies and experimental strategies, recent research has revealed an expanding universe of non-coding RNAs (ncRNAs). The importance of ncRNAs was anticipated by the observation that an organism’s complexity is scarcely correlated with the number of protein coding mRNAs, yet significantly scales with the number of ncRNAs [1]. Hitherto, several ncRNA classes have been identified as essential players in orchestrating and shaping cellular life by participating, inter alia, in the multi-layered surveillance system of gene expression [1,2]. The term gene expression invokes, in the classical view, the process by which the information encoded in DNA is transcribed into a messenger RNA, which is subsequently used as a template for the synthesis of proteins during the translation process. With the discovery of functional ncRNAs, a significant milestone has been achieved by determining the importance of RNA covering roles other than merely mediating genetic information between DNA and proteins, ultimately leading to an expanded view on the central dogma of molecular biology.

The ability to continuously sense and accordingly regulate gene expression upon environmental changes is crucial for all living cells to preserve cellular functions and establish and maintain the necessary homeostasis, adaptation, and stress responses [3]. In this regard, ncRNAs have been revealed to play an essential role in the tight and coordinated control of gene expression at the transcriptional as well as at the post-transcriptional level. Remarkably, diverse studies measuring expression on the genome-wide scale reported the paucity of the correlation between mRNA and protein levels, particularly in multicellular organisms, thus accentuating the importance of regulation at the post-transcriptional level [4,5,6,7]. Indeed, accumulating evidence suggests that the rapid adaptation of the proteome to cellular stress may be principally controlled at the level of translation, one of the final steps in gene expression [8,9]. To date, numerous pathways for the ncRNA-mediated regulation of protein synthesis have been described [10,11]. Some of the most prominent examples include eukaryotic microRNAs (miRNAs) and small interfering RNAs (siRNAs), as well as bacterial *cis*- and *trans*-acting antisense RNAs (asRNAs) [12,13]. Interestingly, the majority of so far documented ncRNAs capable of fine-tuning translation target the mRNA transcripts.

**Figure 1:**
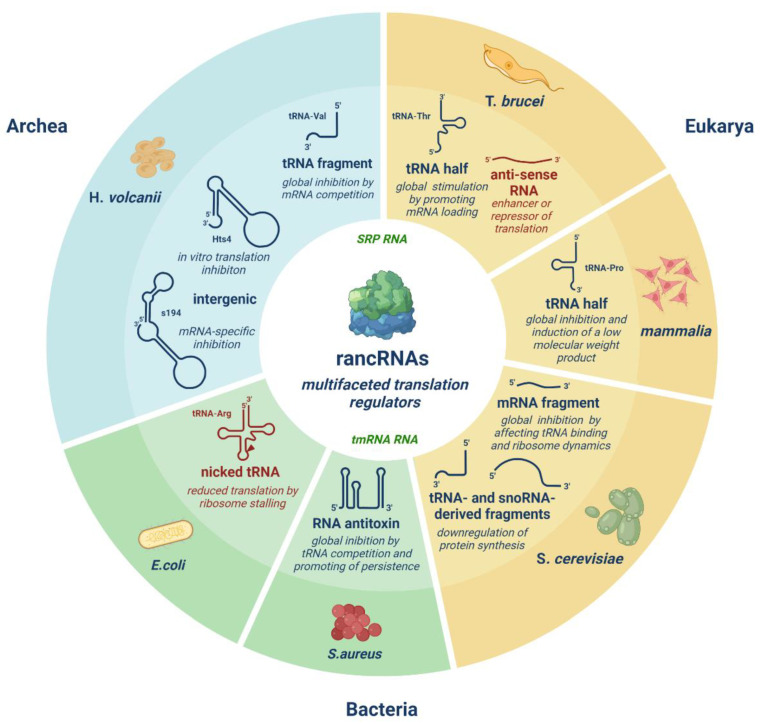
Overview of the expanding class of ribosome-associated ncRNAs (rancRNAs) representing a vast heterogeneous group of riboregulators. RancRNAs differ in their origin, length, and mode of action. RancRNA-mediated translation regulation has been identified in various organisms, spanning all three domains of life. Blue: validated rancRNAs. Red: novel potential rancRNAs. Green: well-characterized rancRNAs (tmRNA in bacteria, SRP RNA almost universally conserved).

However, improved high-throughput RNA sequencing methods recently revealed the ribosome as a novel target for a largely unknown class of non-coding regulatory RNA molecules [14]. The ribosome, a large macromolecular congregation of RNA and proteins, represents the pivotal enzyme of protein synthesis, at the heart of the central dogma. These so-called ribosome-associated ncRNAs (rancRNAs) have emerged in ncRNA research as efficient players in the rapid regulation of translation by directly binding the ribosome in a stress-dependent manner (Figure 1) [15,16,17,18,19,20,21,22]. According to our definition, rancRNAs are transcripts that (i) directly bind to mature ribosomes or ribosomal subunits (as naked RNA or as RNA-protein complex), (ii) lack protein-coding potential, and (iii) affect the efficiency or specificity of protein biosynthesis upon ribosome interaction. RancRNAs constitute a very heterogeneous group of regulatory RNA molecules; they show a vast diversity in their origin, length, and mechanism of action. Some of these ncRNAs have been found to modulate protein biosynthesis on a global scale, while others affect the translation of specific mRNAs, such as, for example, the well described bacterial transfer-messenger RNA (tmRNA) and the almost universally conserved signal recognition particle (SRP) RNA [23,24]. Regarding the mode of action, rancRNAs have been shown to modulate both the initiation and the elongation phases of translation, but in theory, each of the four known steps of the ribosomal translation cycle could be subject to rancRNA-mediated regulation.

The rancRNAs that have been functionally characterized so far either originate from intergenic regions, and thus derive from genuine genes, or are post-transcriptional processing products of functional RNA molecules, such as mRNA, snoRNA, or tRNA. Indeed, accumulating evidence has highlighted that mRNA and tRNA molecules do not solely participate in protein synthesis as genetic templates and carriers of amino acid to the ribosome, respectively, but also have the potential to give rise to general modulation factors of the translational process. There is a vast literature reporting the plethora of small ncRNAs generated by processing events of well characterized RNA molecules. In early studies, these small RNA fragments were considered as functionless degradation products. Currently, there is mounting evidence in all three domains of life supporting that these widespread and likely conserved processing products exert key regulatory functions, including the modulation of protein synthesis [25,26,27]. As a matter of fact, the majority of rancRNAs that have been hitherto identified are processed out of tRNAs [15,17,18,20,21].

One of the most impressive attributes of these novel riboregulators, which often consist of only a few nucleotides, is their ability to exert a David–Goliath-type control over the cell’s giant protein factory. Given the fact that the production of proteins by ribosomes is a highly energy-demanding process, it is subjected to stringent control, which allows gene expression to rapidly change in response to various stresses [28,29]. This immediate reprogramming of protein biosynthesis is crucial for cellular adaptation and survival under conditions of physiological changes [30,31,32]. Indeed, the reduction in global protein synthesis makes it possible to redirect saved energy towards cellular needs. Regarding these aspects, a noteworthy advantage of rancRNAs is their almost instantaneous availability, since they function at the RNA level and thus do not necessitate prior translation into proteinaceous regulator molecules to perform their task. Therefore, rancRNAs are ideal, rapid modulators of cellular response to changing environmental conditions and are considered pivotal in the first wave of stress adaptation [14].

In this review, the results of recent studies on the ribosomal ncRNA interactomes and the heterogeneous functional repertoire of rancRNAs are illustrated. We present several examples of rancRNAs and their putative role in translation regulation. The direct association of specific rancRNAs with the ribosome has been experimentally confirmed in various organisms, spanning all three domains of life (archaea, bacteria, eukaryotes), which has greatly expanded our understanding of the post-transcriptional regulation of gene expression. Considering that the ribosome is highly conserved throughout evolution, rancRNA-mediated translation control is expected to exist to a much greater extent, as initially assumed, thus providing an interesting avenue for future research.

## 2. Methodologies for the Identification of rancRNAs

**Figure 2:**
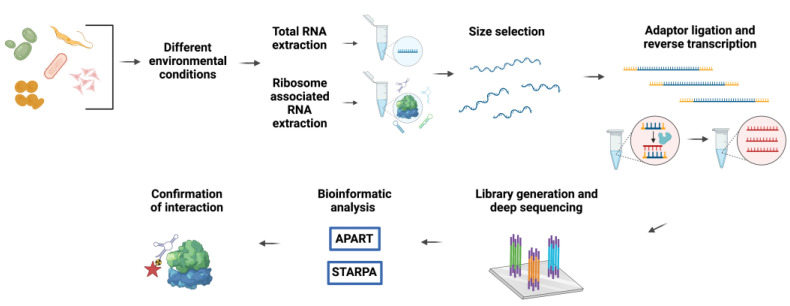
The general workflow adopted for the isolation and identification of rancRNAs.

The initial fortuitous finding of ribosome-bound ncRNAs in polysome-derived RNA-sequencing data [33], which were probably prematurely referred to as ‘contaminants’, has fostered more dedicated research on the ribosomal ncRNA interactome. In the recent past, our laboratory conducted several targeted RNome screens for ribosome-associated ncRNA in various model organisms spanning all three domains of life (Figure 2) [15,20,21,34,35]. To detect novel ncRNAs putatively regulating the ribosome’s activity, various stress conditions were applied to the different model systems. We reasoned that during challenging environmental conditions, situations in which rapid adaptation programs are central, translation regulators, such as rancRNAs, would be of particular significance. Subsequently, the small transcriptome in the size range between 20 and 300 nucleotides was extracted from the total RNA, as well as from the ribosome-bound fraction for each studied condition. The isolated RNomes were then converted into cDNAs for deep-sequencing analyses. The performed studies revealed a vast amount of small and long ncRNAs originating either from intergenic regions of the genome or from processing events of a functional parental RNA, which include mRNAs, tRNAs, snoRNAs, and rRNAs. Moreover, the reported rancRNAs showed stress-specific expression and enrichment in ribosomal and polysomal fractions. One of the major difficulties encountered during the bioinformatics analyses of the deep-sequencing data was to distinguish stable RNA processing products with possible functions from degradation products. This was mainly due to the complexity of RNA processing, which constitutes a multiple-layer process. Hence, a single parental transcript is able to generate several functional ncRNAs by different processing events. To circumvent this problem, novel computational pipelines were developed to identify and quantify stable functional RNA processing products and novel ncRNA transcripts from the obtained RNA-Seq reads. Some examples include: APART (automated pipeline for analysis of RNA transcripts), used to analyze cDNA libraries from *Saccharomyces cerevisiae, Haloferax volcanii*, and *Trypanosoma brucei* [15,16,17,18,19,20,34], and STARPA (stable RNA processing product analyzer), designed for analyses in *Escherichia coli* [35]. Ultimately, the expression, ribosome association, and functionality of the predicted rancRNA candidates, which include novel ncRNA species or processing products derived from diverse primary RNAs, always have to be experimentally verified via, for example, northern blot or qRT-PCR analysis, and approaches testing the effects on ribosome functionality.

## 3. Bacterial rancRNAs

The power of the regulatory effect of rancRNAs on protein synthesis lies in their swift and efficient mode of action, which mostly takes place in response to environmental stress. This mechanism has been shown to be conserved in all domains of life, demonstrating its importance as an adaptation strategy that was preserved during evolution.

In the bacterial domain, two examples of rancRNAs have been known for a long time and have been extensively studied. First, the transfer-messenger RNA (tmRNA), found exclusively in bacteria, ensures that the protein synthesis capacity of the cell is maintained by rescuing ribosome stalling, such as at truncated mRNAs lacking a stop codon, through the trans-translation mechanism [36]. The second example, also present in the other two domains of life, is the signal-recognition particle RNA, an ncRNA component of the signal-recognition particle (SRP) ribonucleoprotein complex. The SRP complex recognizes a specific signaling peptide chain of transmembrane proteins emerging from the ribosomal peptide exit tunnel. Upon the recognition of the signal sequence by the SRP on the ribosome, translation is arrested. After this temporary pause, the SRP is able to target the ribosome-nascent chain complex to membrane translocation sites [37].

In order to investigate the RNA’s post-transcriptional regulatory network in bacteria, our laboratory conducted a study on the small transcriptome (20–300 nts) of exponential and stationary phases *Escherichia coli*, with the focus on total cellular small RNA and ribosome-associated RNAs. Numerous small ncRNA candidates were identified, showing different expression between the two growth phases. Furthermore, the ribosome-bound deep-sequencing data indicated the possible interaction of several rancRNAs with *E. coli* ribosomes. Further investigations are necessary to validate these interactions and to potentially assign a functional role to these putative rancRNA candidates [35].

Recently, a rancRNA candidate was discovered in *E. coli* colicin-D-sensitive cells. Colicin D is a bacteriocin that targets tRNA^Arg^, preferentially tRNA^Arg^_ICG_, and cleaves it at the 3′ of the anticodon loop, forming nicked tRNA^Arg^_ICG_ [38]. This cleavage results in translation impairment in vitro and reduced cell viability. Ogawa et al. observed that this effect was not induced by a decreasing amount of intracellular level of tRNA^Arg^_ICG_, but instead by the accumulation of nicked species. This indicates that the cleaved form of tRNA^Arg^_ICG_ has an active role in attenuating protein synthesis. Moreover, they demonstrated that the cleaved tRNA^Arg^_ICG_ was still recognized by the prokaryotic translation elongation factor EF-Tu and able to form the tertiary complex. The model proposed by the authors suggests that cleaved tRNA^Arg^_ICG_ inhibits translation by transiently occupying the A-site of the ribosome and causing ribosome stalling. This scenario would designate nicked tRNA^Arg^_ICG_ as rancRNA [38].

Another rancRNA functionally characterized in bacteria is the type I RNA antitoxin SprF1, expressed by the human pathogen *Staphylococcus aureus*. Besides its well understood role as an antisense regulator of the SprG1 toxin mRNA, SprF1 is a dual-functional ncRNA [22]. Indeed, it interacts with ribosomes and polysomes at the subunit interface through the purine-rich sequence present at the 5′-end of SprF1. In vitro and in vivo evidence demonstrated that this interaction has a regulatory nature and attenuates protein synthesis in a dose-dependent manner. The translation step affected by this rancRNA seems to be the initiation phase. Indeed, the interaction of SprF1 with the ribosome drastically decreases mRNA loading by competition and reduces tRNA binding to the ribosomal P-site. Moreover, the data obtained in this study indicated that under hyperosmotic stress, the expression level of SprF1 significantly increases, together with its enhancement of stability and accumulation on the ribosome. Pinel-Marie et al. finally demonstrated that, by inhibiting global translation, the SprF1 antitoxin facilitates the formation of persistent *S. aureus* cells, resulting in the possible development of general antibiotic tolerance [22].

## 4. Archaeal rancRNAs

The archaeal species most utilized so far in dedicated RNome screens for rancRNAs is *Haloferax volcanii*, a halophilic archaeal model organism. From the deep-sequencing analysis of small ncRNAs interacting with ribosomes isolated from *H. volcanii* cells, grown in normal and environmental challenging conditions, numerous rancRNA candidates emerged [15]. In total, 26% of the total reads consisted of tRNA fragments (tRFs). These fragments originated almost exclusively from the 5′ end of different mature tRNAs. Experimental evidence demonstrated that most of these tRFs bind to the ribosome in vitro and in vivo [15].

One tRF stood out for its abundance in the screen, namely a 26-nucleotide long fragment originated from the 5′ end of the tRNA^Val^_GAC_. This Val-tRF was processed specifically during alkaline stress conditions and, upon ribosome association, showed global translation inhibition [15]. Val-tRF binds to the 16S rRNA of the small ribosomal subunit, more precisely, to a region in close proximity to the mRNA channel. From a mechanistic point of view, Val-tRF’s biological role consists of inhibiting translation initiation by competing with mRNA for binding to the small ribosomal subunit [18]. However, this appears not to be the only way in which Val-tRF affects translation. Indeed, in the presence of this rancRNA, peptide bond formation is also inhibited, at least under in vitro conditions [18]. Thus, these findings suggest a potentially additional role for Val-tRF during the elongation phase of protein synthesis.

The recently characterized archaeal rancRNA, rancRNA_s194, was shown to be involved in the regulation of the translation elongation phase of *H. volcanii* [15,19]. Hitherto, this has been the only rancRNA proven to regulate the translation of a specific mRNA by directly interacting with the ribosome. Wyss et al. demonstrated that rancRNA_s194 is abundantly expressed in the exponential phase, associates predominantly with polysomes, and is localized to the binding site on the large subunit [19]. The deletion strain of this ncRNA showed a shorter lag phase and faster growth rates in media containing non-standard sugars, such as sole carbon source. This suggested that this rancRNA indeed has a regulatory role. However, in contrast to what was shown for all the other rancRNAs studied so far, rancRNA_s194 was not able to perturb global translation in vivo. A proteomic analysis, carried out to pinpoint the source of the growth phenotype, revealed the carbon starvation protein CstA to be upregulated upon rancRNA-s194 deletion. This protein, a peptide transporter, is generally upregulated in cells facing nutrient-limiting conditions. In other organisms, the expression of CstA is negatively regulated at the translational level by the protein CsrA, which is not present in *H. volcanii*. It therefore appears reasonable to suppose that rancRNA_s194 replaced CsrA as a regulator of the expression of CstA. Further studies are required in order to elucidate the mechanism by which rancRNA_s194 regulates the expression of a specific mRNAs by binding the ribosome. This will provide new insight into gene expression regulation by introducing a new mechanism for mRNA-specific control that is different from RNAi.

Alongside rancRNA_s194, another rancRNA candidate was identified in this study [15]. This intergenic ncRNA, named Hts4, strongly interacts with *H. volcanii* polysomes in exponentially growing cells [19]. In vitro experiments reported an inhibitory effect on translation, which suggests a possible active role of Hts4 in the modulation of global translation in vivo. However, subjecting the Hts4 knock-out strain to different stress conditions did not result in a growth phenotype. Additional investigations are necessary to understand the mechanism by which Hts4 attenuates translation and in which physiological context it exerts this role [19].

A recent computational transcriptomic analysis performed in the organism *Halobacterium salinarum* hinted at the presence of the rancRNA regulatory mechanism in this archaeon. In this study, de Almeida et al. mapped the primary anti-sense transcriptome of *H. salinarum* and, comparing these data with the public Ribo-seq (ribosome profiling) data, they identified 91 significant anti-sense RNAs associated with the ribosome [39]. De Almeida et al. affirm that this interaction might suggest either a coding potential or the possibility of a regulative role for these RNAs as rancRNAs. These computational data still require experimental support but provide a starting point for future studies [39].

## 5. Eukaryal rancRNAs

*S. cerevisiae* is one of the most intensively characterized eukaryotic model organisms. It lacks the post-translational regulation of RNA interference, thus living without miRNA and siRNA and is therefore an ideal model system for uncovering so far unknown ncRNA-based regulation beyond classical RNAi [40].

In 2012 the first transcriptome-wide screen for rancRNAs was performed in *S. cerevisiae* under normal conditions and following environmental stress [34]. Despite its limited sequencing depth compared to contemporary RNome screens, this pilot study resulted in the identification of about 130 small rancRNA candidates in a size range between 20 and 300 nts. The origin of these ncRNAs was extremely heterogeneous, spanning from intergenic regions of the genome to the processing product of functional RNAs, such as mRNAs, tRNAs, snoRNAs, SRP RNAs, and rRNAs. A large number of these ncRNA candidates showed a stress-related expression [34].

One of the most abundant new ribosome interactors that resulted from this first screen was rancRNA_18, an mRNA exon-derived ncRNA which is also the most characterized rancRNA so far [16].It was found as part of the rancRNAs with a stress independent expression pattern, but it turned out to regulate translation in a hyperosmotic-stress-related manner. This 18-nt-long fragment originates from the *TRM10* locus, which encodes for a tRNA methyltransferase. The deletion strain of *TRM10,* and thus also of the genomically embedded rancRNA_18, showed a severe growth inhibition phenotype specifically in hyperosmotic stress conditions. The expression of rancRNA_18 is not locus-specific, since expression from a different yeast chromosome in the *TRM10* knock-out strain could fully complement the phenotype, thus demonstrating rancRNA_18 to be a genuine ncRNA whose gene was embedded in the *TRM10* open reading frame during the course of evolution [41]. Furthermore, Pircher et al. demonstrated that the interaction of rancRNA_18 with the ribosome was sequence-specific [16]. A particular effort was made in order to identify the enzyme responsible for the rancRNA_18 processing from the *TRM10* mRNA. The secondary structure prediction of this mRNA-derived fragment and biochemical evidence support the idea that the endonuclease Rnt1, known for its role in yeast rRNA maturation, is the enzyme involved in processing of rancRNA_18 [41]. In vitro and in vivo studies demonstrated that rancRNA_18 binds the ribosome and attenuates global translation during the early stages of harsh hyperosmotic stress. This fast response to environmental change provides the cell with a sufficient time span to adopt stress-specific strategies to survive [14,41]. rancRNA18 is also the first ribosome-bound ncRNA for which a cryo-EM structure was published [41]. In combination with numerous biochemical data it was possible to describe the mode of action of this ribosome bound ncRNA. rancRNA_18 binding site was identified in the large ribosomal subunit close to the E-site tRNA region, causing a loss of flexibility in the L1 stalk region. The highly dynamic L1 stalk is known for its involvement in the E-tRNA ejection from the ribosome. Thus, freezing the ribosome in this non-productive state is incompatible with the efficient tRNA release needed for effective and consecutive rounds of tRNA translocation during the elongation phase of protein biosynthesis. Moreover, the rancRNA_18-mediated inhibition of ribosome dynamics also affected A-site tRNA binding affinity, which was significantly reduced in the presence of ribosome-bound rancRNA_18. Taken together, the available biochemical, genetic, and structural data suggest that rancRNA_18 is a global translation attenuator relevant at the onset of hyperosmotic stress that affects ribosomes at early stages of the ribosomal elongation cycle and, thus, immediately after translation initiation [41].

In the same transcriptome analysis, 41 tRNA-derived fragments (tRF) were identified interacting with the yeast ribosome [34]. Out of these tRFs, 26 derived from the 3′ half and the rest from the 5′ half of different tRNAs. In vitro and in vivo experiments showed that both 5′-tRFs and 3′-tRFs from *S. cerevisiae* were able to bind the ribosome and regulate global translation [17]. The binding site for these tRFs seems to not overlap with the three tRNA binding sites, since no competition with full-length tRNA was observed. On the other hand, all tested tRFs competed for the ribosome binding site, suggesting that they all occupy the same spot or that different binding sites overlap with each other. Between all the tRFs studied, both 3′ and 5′-tRFs-His showed the highest regulatory potential during amino acid starvation stress, inducing the downregulation of protein synthesis [17].

Further studies suggested an interaction between some of the yeast aminoacyl-tRNA synthetase with the ribosome and that putative tRFs rancRNAs might negatively modulate protein biosynthesis indirectly via affecting aminoacylation by interacting with this ribosome-associated aminoacyl-tRNA synthetase [42].

Another class of ncRNAs that emerged from the initial yeast rancRNA screen was the snoRNAs and snoRNA-derived fragments (sdRNAs), which result from the processing of mature snoRNAs [34]. SnoRNAs are known for their role in the nucleolus for guiding modification enzymes to maturating rRNAs [43]. More recently, non-canonical functions of soRNAs, independent of ribosome biogenesis, have also been described. Until recently, the only non-canonical activity concerning the translation regulation of these molecules was limited to a miRNA-like function of some snoRNA fragments in human cells [44]. The localization of snoRNA-derived fragments on the ribosome, in an organism lacking the miRNA pathway such as *S. cerevisiae*, suggests a new regulatory role for these processing products. It could be shown that snoRNA-derived fragments are present in the cytoplasm [34] where they associate with the ribosome in a stress dependent manner leading to inhibition of translation in vivo and in vitro [43]. However, for this last study it is important to take into consideration the limitation represented by the very low amount of snoRNAs and sdRNAs detected.

The ability of rancRNAs to confer cellular resilience recently found confirmation in other eukaryotic model systems, such as the human pathogenic parasite *Trypanosoma brucei*. In contrast to other eukaryotes, this organism largely lacks transcriptional control of individual genes because of its unusual eukaryal polycistronic gene organization [45]. Thus, the regulation of gene expression occurs mainly on the post-transcriptional level. Deep sequencing of the small non-coding RNA interactome of ribosomes under stress and unstressed conditions in the parasite *T. brucei* disclosed the first rancRNA exerting a stimulating function on global translation [20]. Among different putative ribosome-associated tRNA fragments, the tRNA^Thr^ 3’ half was found to be one of the most abundant tRFs during starvation and in late stationary phase. During the stress recovery phase of procyclic *T. brucei* cells from starvation, the tRNA^Thr^ 3′ half has been shown to associate with ribosomes and polysomes and to promote protein synthesis both in vitro and in vivo. From a mechanistic point of view, the first in vitro experiments hint that the tRNA^Thr^ half enhances translation initiation by facilitating mRNA binding to the ribosome. An analogous stimulation in translation activity was observed after introducing the *T. brucei* tRNA^Thr^ half in *H. volcanii, S. cerevisiae*, and HeLa, suggesting a highly conserved mode of action throughout evolution [20]. Moreover, our current studies revealed the site of biogenesis and action of the tRNA^Thr^ 3′-half in the mitochondria of *T. brucei* (unpublished data). Thus, the available data indicate that this tRNA half is generated upon nutritional stress in the single mitochondrium of the parasite, where it associates with the large ribosomal subunit of the mito-ribosome to boost protein production inside the central organelle of energy metabolism during stress recovery (our unpublished data).

In the recent past, two novel potential trypanosomal rancRNAs have been described: the non-coding anti-sense RNA regulators TBsRNA-33 and TBsRNA-37 [46]. These two anti-sense regulators are several hundreds of nucleotides long and originate from rRNA loci and have been shown to co-fractionate with the 80S ribosome and regulate translation of the mRNAs they interact with [46]. TBsRNA-33 was revealed to be an anti-sense repressor of translation. One interesting target of TBsRNA-33 that has been focused on is the Pif1 mRNA [46]. The encoded protein, DNA repair and recombination helicase protein PIF, is fundamental for the maintenance of the mitochondrial genome [47]. The translation repression of Pif1 mRNA by the ncRNA TBsRNA-33 suggested the putative involvement of TBsRNA-33 in the regulation of a specific mitochondrial mRNA translation. On the other hand, TBsRNA-37 has a putative role as an anti-sense enhancer of translation and potentially base-pairs with mature rRNA as well [46]. Although experimental validation of the direct association to the ribosomes is pending, these two anti-sense RNAs can be envisioned as potential novel members of the repertoire of trypanosomal rancRNAs.

Very recently, the research into novel rancRNA expanded to mammalian systems. A highly conserved tRNA^Pro^ 5’ half was identified through screening for ribosome-bound tRNA-derived fragments in different mammalian cell lines, including CHO-K1, HeLa, HEK, BON, NCI, and Hep3B cells [21]. Experimental evidence reported the association of the 35 nucleotides long tRNA^Pro^ 5′ half with ribosomes and polysomes in several mammalian model systems. Moreover, an in vitro experiment showed the downregulation of global translation upon the addition of this tRNA-derived rancRNA. The tRNA-^Pro^ 5′ half crosslinked to the small ribosomal subunit rRNA close to the subunit interface side. Concomitantly with the inhibitory effect on protein synthesis, the induction of a specific low-molecular-weight product has been observed in vitro and in vivo. This accumulating ~17 kDa side product was shown to be sensitive to protease K digestion and RNase I treatment, suggesting that it consists of both protein and RNA components. Further analysis revealed this tRNA^Pro^ 5′ half-dependent by-product to be peptidyl-tRNA that likely accumulated during rancRNA-mediated ribosome stalling [21].

In human cells, tRFs derived from 5′ end of tRNAs were found to inhibit protein translation in a sequence-independent manner [48]. A fraction of 5′ tRFs has been observed to partially co-migrate with translating ribosomes in density gradients [48]. Interestingly, 5′ tRFs have been shown to modulate protein synthesis without requiring complementary target sites in the mRNA transcript, but instead depend on a so called “GG” dinucleotide motif that appears to be conserved among all 5′ tRFs [48]. However, a follow-up study showed that the identified tRNA-derived fragments modulated translation by associating primarily with the human multisynthetase complex (MSC). Moreover, the GG-dinucleotide motif has been observed to be pivotal to the interaction with the MSC [49]. This recent finding lessened the possibility of a direct interaction of these tRFs with translating ribosomes and thus challenged the classification of these particular tRFs as rancRNAs.

The deep-sequencing data of the small RNA transcriptome in *Drosophila* embryonic extracts uncovered the temporal and selective expression of numerous tRFs originating preferentially from the 5′ end sequences of a subset of tRNAs [50]. Although most of the tRFs were found primarily in the mRNP fraction, a small portion of tRNA fragments co-sedimented with monosomal and polysomal fractions, thus representing potential novel rancRNAs in insects [50].

In *Arabidopsis thaliana*, two 20-nucleotide-long tRFs derived from the 5′ end of tRNA^Ala^ and tRNA^Asn^ were shown to efficiently attenuate translation in vitro [51]. Moreover, Arabidopsis tRF^Ala^ was found in heavy polysomal fractions [51], suggesting that tRFs potentially participate as rancRNAs in the regulation of gene expression in plants.

## 6. Ribosome-Bound Long ncRNAs

The vast majority of the mammalian genome is transcribed into RNAs lacking protein coding potential [52,53]. A subclass of these transcription products is referred to as long non-coding RNAs (lncRNAs), which are defined as transcripts longer than 200 nucleotides that are not translated into proteins and are localized to both the nucleus and the cytoplasm [54]. LncRNAs show properties common to conventional mRNA transcripts, which comprise transcription by RNA polymerase II, a 5′-end 7-methylguanosine (m7G) cap, and polyadenylation of the 3′ end [55].

Growing evidence showed that these widely expressed ncRNAs perform key roles in gene expression regulation at multiple levels [56,57]. For instance, lncRNAs are implicated in the regulation of chromatin structure and function, and orchestrate RNA transcription, splicing, stability, and translation. The utilization of state-of-the-art techniques such as ribosome profiling, ribosome fractionation, and translating ribosome affinity purification (TRAP) have recently unveiled the association of numerous cytoplasmic lncRNAs with the ribosome and polysome fractions [33,58,59,60,61,62,63,64,65,66,67,68]. These findings imply the possibility that some of these cytoplasmic lncRNAs may function as modulators of ribosome activity and, thus, would represent genuine rancRNAs according to our definition.

However, lncRNAs’ co-sedimentation with ribosome and polysome might also be explained by other means. Contrary to their general definition, several lncRNAs were found to possess small open reading frames (smORFs) with pronounced ribosome occupancy [69,70,71]. Multiple experimental data gathered during the past two decades confirmed that several smORFs embedded in lncRNAs code for functional small proteins or micropeptides with fundamental biological functions [72,73,74,75,76,77,78,79,80,81,82]. Thus, strictly speaking, these lncRNA transcripts no longer fulfil the underlying definition of non-coding RNAs and, therefore, would also not qualify as canonical rancRNA candidates. On the other hand, the association of some lncRNAs that indeed lack open reading frames with ribosomes might very well belong to the novel class of rancRNA molecules.

For the possible classification as rancRNA, it is decisive to discern whether lncRNAs bind directly to ribosomes. LncRNAs can activate or repress the translation of target mRNAs through the formation of RNA–RNA hybrids [64,83,84,85]. In this case, the association with translating polysomes is not direct but is instead mediated by the binding with mRNAs indirectly. Alternatively, the association with ribosomes might be due to the translation-dependent degradation of lncRNAs [63]. For instance, it has been shown that the spliced mammalian lncRNA *Growth Arrest-Specific 5* (GAS5), which contains numerous stop codons, is subjected to nonsense-mediated decay (NMD) [86,87]. Recent studies investigated a variety of features displayed by lncRNAs that could further explain their association with ribosomes. For example, mRNA-like features at the 5′ end, such as long “pseudo-5′UTRs” and a 5′ methyl-guanosine cap, could contribute to recognition by ribosomes, as in the case of mRNA [63,88]. The recognition of the 5′ end is crucial not only for translation initiation, but also for nonsense-mediated decay (NMD) [89]. However, the translation of lncRNAs can also initiate in a cap-independent manner. For example, head-to-tail joined circular RNAs (circRNAs), which lack 5′ caps and poly(A) tails, have been shown to associate with translating ribosomes and were proposed to have the potential for encoding proteins by using internal ribosome entry sites (IRESs) [90,91,92]. Moreover, recent studies showed that the translation of circRNAs is facilitated by 18S rRNA complementarity and structured elements containing IRES [93]. However, the possibility cannot be excluded that ribosome-associated circRNAs may have an unknown regulatory function on the translation machinery.

Overall, a complete understanding of the role of ribosome-bound lncRNA remains elusive. Arguably one of the most intriguing hypotheses concerning lncRNAs’ association with the ribosomes is that lncRNAs actively participate in regulating translation by targeting the ribosome directly. Thus, the role of polysome-associated lncRNAs merits further investigation.

## 7. Conclusions and Perspectives

**Figure 3:**
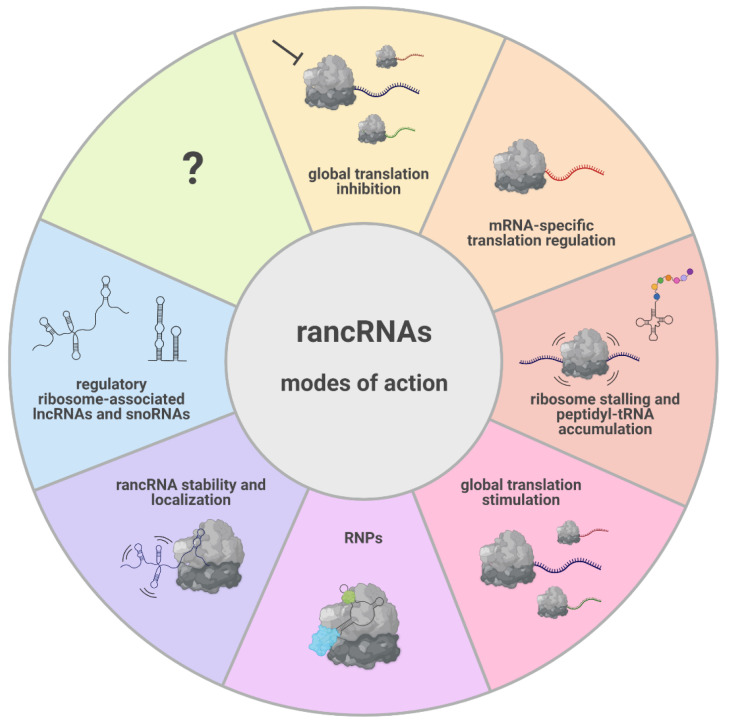
The diverse modes of action of rancRNAs and the functional consequences of their interaction with the ribosome. RancRNAs can exert their biological role as naked RNA molecules or as part of RNP complexes (e.g., SRP). The binding of rancRNAs to the ribosome can influence their stability. The origin and length of rancRNAs is extremely diverse, spanning from lncRNAs to very small RNA fragments. The interaction with the ribosome can modulate translation on a global level; the interaction is able to inhibit (e.g., yeast rancRNA_18) and stimulate (e.g., Trypanosomal tRNA^Thr^ 3′ half) translation, target the expression of specific mRNAs (e.g., archaeal rancRNA_s194), or cause ribosome stalling and peptidyl-tRNA accumulation (mammalian tRNA^Pro^ 5′ half).

Over the past decade, mounting evidence has confirmed ribosome-associated ncRNAs to be involved in translation modulation, representing a molecular strategy conserved in all three domains of life. This class of ncRNAs is characterized by the extremely heterogeneous nature of the molecules with regard to the biogenesis, length, and mode of action (Figure 3). Indeed, rancRNAs can originate from intergenic regions of the genome or represent processing products of functional coding and noncoding precursor transcripts. This heterogeneity also applies to the effect that rancRNAs have on protein production, being able to act as global regulators of protein synthesis, and therefore capable of affecting the entire proteome, or to directly modulate the expression of individual mRNAs. They can target ribosomes engaged in the initiation as well as the elongation phase of protein synthesis, inhibiting or enhancing translation. On the other hand, they all seem to share the same purpose: helping cells to overcome the stress induced by challenging environmental conditions. For this purpose, rancRNAs can provide an efficient and fast response that is also energetically inexpensive for the cell. In contrast to what once was thought to be degradation material co-purifying with ribosomes, rancRNAs have been uncovered in the past years as potent regulators of the translation machinery.

Although the physiological importance of rancRNAs in translation control has since been recognized in different model systems, many questions still remain to be answered. Most importantly, the mechanism of action has so far only been described for a small number of rancRNAs in molecular detail. Thus, dedicated functional research, ideally aided by high-resolution structural studies, is required to address the burning question of how such small RNA molecules can orchestrate the efficiency and specificity of the giant ribosome. Furthermore, since rancRNAs appear to be involved in the first wave of stress response, in many cases it is still unclear if and how the expression of rancRNAs is regulated at the transcriptional level or at the level of post-transcriptional processing. Similarly, virtually nothing is known about the mechanism of rancRNA inactivation or turnover once the challenging stress situation ceased. It is possible, but by no means clear, that rancRNA-binding proteins could play crucial roles in these processes. Thus, another pivotal milestone for understanding rancRNA-mediated translation regulation is the identification of protein-binding partners. Future studies will be necessary to deeply investigate rancRNAs not only from a mechanistic point of view, but also for their broad applications. The identification of such regulators in human pathogens could provide new insights into drug resistance strategies adopted by prokaryotes and could contribute to the development of RNA-based therapies against microbial infections [22,94]. Moreover, recent findings demonstrated the implication of tRNA-derived fragments (tRFs) in human diseases, such as cancer [95]. Since an increasing number of tRFs appear to be rancRNAs, they might affect the rate of protein biosynthesis during tumorigenesis. Therefore, future research on the role of human rancRNAs has the potential to offer new perspectives on cancer biology and treatment strategies. In conclusion, rancRNAs represent a new class of regulatory molecules that are still to be widely explored, yet already show exceptional potential.

## Data Availability

Not applicable.
